# Reduction and Control Technology of Harmful Dicarbonyl Compounds in Flounder (*Pleuronectiformes*) Seafood Condiment Preparation

**DOI:** 10.3390/foods14101717

**Published:** 2025-05-12

**Authors:** Fazhao He, Yinggang Ge, Hui Chen, Shanyu Wang, Deqing Zhou, Mingchao Pan, Rong Cao, Guohui Sun

**Affiliations:** 1 College of Food Science and Engineering, Ocean University of China, 1299# Sansha Road, West Coast New District, Qingdao 266003, China; 2 Yellow Sea Fisheries Research Institute, Chinese Academy of Fishery Sciences, 106# Nanjing Road, Shinan District, Qingdao 266071, China

**Keywords:** α-dicarbonyl compounds, Maillard reaction, Flounder, seafood condiment, reduction technology

## Abstract

Harmful α-dicarbonyl compounds (α-DCCs) were formed via Maillard reaction (MR) during the production of seafood condiments. The method of reducing α-DCCs could be achieved through optimizing the MR parameters. In this study, Flounder (*Pleuronectiformes*) steak was chosen as the raw material for developing seafood condiments with lower α-DCCs using liquid chromatography–tandem mass spectrometry (LC-MS/MS). Indicators such as amino acid nitrogen, peptides, and total antioxidant capacity (T-AOC) of enzymolysis hydrolysates were applied to evaluate the enzymolysis effects on Flounder steak in different protease groups. When optimizing the parameters in MR, an optimal formulation with lower α-DCCs was chosen from the flavourzyme group to prepare Flounder seafood condiment at 105 °C, pH 6.5, 1.5% D-xylose addition, and a 20 min reaction time. The concentrations of methylglyoxal (MGO), glyoxal (GO), 2,3-butanedione (2,3-BD), and 3-deoxyglucosone (3-DG) were reduced to 1.23, 0.23, 0.01, and 0.05 μg/g, respectively, which were lower than those identified in 10 commercial seafood condiments (1.84, 0.39, 0.09, and 0.05 μg/g) and conformed to the standards of daily intake in the United States and the European Union. The quality verification demonstrated that the optimal Flounder seafood condiment had a similar odor profile but with higher intensity than that of the products on the market, which scored 89.79 in sensory evaluation. The results indicated that the process optimized in this study could be applied to prepare a Flounder seafood condiment with lower α-DCCs. This processing technology to control α-DCCs may be employed to improve the quality and safety of foods and contribute to human health.

## 1. Introduction

Seafood condiments have unique flavors and high quality, which are essential to their continued usage in human daily life [1,2]. In the production of seafood condiments, except for adding rich proteins, amino acids, and other nutrients obtained from aquatic products [3,4], the Maillard reaction (MR), as a necessary process, plays an important role in quality improvement. However, along with the production of flavor substances, α-dicarbonyl compounds (α-DCCs) affecting food consumption safety are formed through the auto-oxidation of glucose and the degradation of Amadori rearrangement products and deoxy sugars. Therefore, reducing the α-DCC contents of seafood condiments has become a key technology in quality control.

α-DCCs are the vital intermediates of the MR, with a high reaction capability and risk to human health [5]. Meanwhile, α-DCCs had a cumulative effect in the organism, and it was found that about 1/3 of them could not be metabolized and excreted from the body [6]. Moreover, α-DCCs such as methylglyoxal (MGO), glyoxal (GO), 2,3-butanedione (2,3-BD), and 3-deoxyglucosone (3-DG) could react with amino acids, peptides, proteins, and phospholipids to generate advanced glycation end products (AGEs), which contribute to underlying chronic diseases including cardiovascular disease, kidney disease, diabetes, and liver disease [7,8,9,10]. The accumulation of α-DCCs is now known to be detrimental to human health and has attracted widespread attention [7]. Increasingly effective methods have been applied to research the contents of α-DCCs in foods with the optimization and application of detection methods. Among them, liquid chromatography–tandem mass spectrometry (LC-MS/MS) has become the main way of detecting α-DCCs due to its high sensitivity, strong selectivity, high separation degree, and ability to simultaneously test the retention time and molecular weight of the analyte. Through the quantification by mass charge ratio of the target material, Tolgahan et al. detected 3-DG, MGO, GO, and 2,3-BD in the processing of biscuits with different raw materials through LC-MS/MS, in which the analysis time was 15 min [11]. Aktağ et al. researched the possible degradation pathway of α-DCCs in fruit juice during storage using LC-MS [12]. Zhu et al. reduced GO and MGO to decrease the formation of odors in pasteurized milk via the LC-MS/MS method [13]. As this demonstrates, the LC-MS/MS method, as a stable quantification method, is widely used in the detection of α-DCCs.

Flounder (*Pleuronectiformes*) is an economically significant fish in temperate waters, and its production has been steadily increasing for decades. Nevertheless, lots of residual substances like fish skin and bones are abandoned in the filleting process, which results in environmental pollution and resource waste. Previous studies have found that Flounder bones comprised about 40% of the weight of fish [14], and Flounder by-products were low in fat and high in protein, DHA, and various minerals [15]. Utilizing the enzymatic hydrolysis of Flounder steaks as a food additive for seafood condiment preparation to enhance the product freshness has become a feasible strategy [16]. But MR remains the main pathway for the generation of α-DCCs. It has been found that the temperature, time, pH, and MR products have a significant influence on MR in complex food-processing systems [17]. Consequently, optimizing the parameters of MR, such as temperature, time, pH, and reducing sugar contents, might offer an effective method to reduce the contents of α-DCCs in seafood condiment preparation.

In this study, the LC-MS/MS method was validated for quantifying 2,3-BD, MGO, and GO. Flounder steak was used as the raw material to prepare enzymolysis hydrolysates with alcalase, flavourzyme, papain, neutrase, and acid protease. Indicators such as amino acid nitrogen, peptides, and total antioxidant capacity (T-AOC) of enzymolysis hydrolysates were applied to evaluate the enzymolysis effects. The MR parameters, including temperature, time, pH value, and D-xylose content, were optimized to reduce the concentrations of 2,3-BD, MGO, GO, and 3-DG in MR products. The contents of four α-DCCs in 10 common seafood condiments were measured as the control to select an optimized formulation for Flounder seafood condiment preparation. New technology to optimize the processing used for reducing 2,3-BD, MGO, GO, and 3-DG was employed to improve the quality and safety of seafood condiments. It provided a feasible way of controlling the α-DCC contents in food. But, at the moment, this detection needs to be performed via LC-MS/MS in the laboratory.

## 2. Materials and Methods

### 2.1. Chemical Reagents and Materials

Fresh raw Flounder was purchased from the local market in Qingdao, China. 2,3-Butanedione (2,3-BD, ≥99.00%) was purchased from Shanghai Macklin Biochemical Co., Ltd. (Shanghai, China). Methylglyoxal (MGO) solution (~40%) was obtained from ALTA Scientific (Tianjin, China). Glyoxal (GO) solution (~40%), 3-deoxyglucosone (3-DG), and O-phenylenediamine (OPD, 99.50%) were supplied by Sigma-Aldrich (Shanghai, China). HPLC-grade methanol (chromatographically pure) was acquired from Oceanpak (Gothenburg, Sweden). All other chemicals were of analytical grade and sourced from Yingrui Chemical (Qingdao, China).

Ten representative seafood condiments, purchased through online and offline sales channels, were selected as the control to evaluate the reducing effects of four α-DCCs (2,3-BD, MGO, GO, and 3-DG) in this study. The selected products included HT oyster sauce, FQM abalone juice, FQM fish sauce-1, HT seafood soy sauce, XH light salt oyster soy sauce, FMZ shrimp paste, SMM Thai fish sauce, FQM fish sauce-2, LJJ abalone juice condiment, and TTL abalone juice oyster sauce. All samples were stored at 4 °C until further analysis.

### 2.2. α-DCCs Analysis

A total of 2.00 g (or 2.00 mL) of a seafood condiment sample was mixed with 8.00 mL of methanol in a centrifugal tube. After vortexed mixing, the mixture was centrifuged at 10,000× *g* for 15 min at 4 °C. Then, 500.00 µL of OPD (10.00 mg/mL) was added to the supernatant for derivative reaction under dark conditions in a 60 °C water bath shaker. The derivatives of 2,3-BD, MGO, GO, and 3-DG were separated by high-performance liquid chromatography (1290 Infinity Ⅱ, Agilent, Waldbronn, Germany) and detected using a 6460 TQ LC/MS system in ESI-positive multiple reaction monitoring mode after purification through a 0.45 μm filter and solid-phase extraction column (Poly-Seri HL B-Pro). The mobile phase consisted of aqueous phase A (deionized 0.10% (*v*/*v*) formic acid in water) and organic phase B (acetonitrile 0.10% (*v*/*v*) formic acid), delivered at a flow rate of 400.00 µL/min. Gradient elution was employed for sample detection as follows: 0–18 min, 20–55% B; 18–20 min, 55–20% B; 20–25 min, 20% B balance. The injection volume was 5.00 µL, and the column temperature was maintained at 25 °C. The retention times of 2,3-BD, MGO, GO, and 3-DG were 9.875, 9.086, 8.141, and 4.834 min, respectively, while their corresponding stable isotopes eluted at 9.893, 9.071, 8.127, and 4.855 min, respectively. The characteristic transitions of parent ions to product ions (Q1 > Q3, *m*/*z*) for 2,3-BD, MGO, GO, and 3-DG were 159.1 > 77.2, 145.1 > 77.2, 131.1 > 77.2, and 235.1 > 199.1, respectively. Qualitative and quantitative analyses were performed using Agilent MassHunter software (V4.1, SCN 644, Waters, Milford, CT, USA).

### 2.3. Proximate Composition and Measurement Analysis of Flounder Steak

The moisture content of the sample was determined by drying in a hot air oven at 105 °C to a constant weight [18]. The ash content was calculated through placing the sample in a pre-weighed porcelain crucible and heating it to 550 °C in a muffle furnace for 6 h [18]. The crude protein content was measured using the Kjeldahl method (6.25 × N) [19]. The lipid content in Flounder steaks was tested according to the reference with slight modifications [20]. The carbohydrate content (CHO) was estimated by difference subtracting the percentage contents of moisture, ash, lipid, and protein from 100.

### 2.4. Preparation of Flounder Polypeptide Lyophilized Powder

Fresh Flounder steaks with bones were processed at 125 °C, 0.25 MPa for 40 min. Subsequently, the Flounder steaks were mechanically chopped and homogenized using a blender (HX-PB1252, Foshan, China). After being rinsed three times with purified water, the obtained Flounder surimi was applied to prepare enzymolysis solutions by adding 400.00 mL of purified water and 5000 U/g of neutrase, acid protease, papain, flavourzyme, and alcalase, respectively, in a water bath (SHA-C, Beijing, China) maintained at 50 °C for 4 h. A 5 min boiling water bath was then applied to inactivate the enzyme. After centrifugation (Neofuge 15R, Shanghai, China) at 6000 rpm for 10 min, the supernatant was freeze-dried (FD5-series, Beijing, China) under vacuum conditions to obtain Flounder crude polypeptide lyophilized powder [21]. The lyophilized powder of crude peptides was stored at −20 °C until further analysis.

### 2.5. Characteristics of Enzymatic Hydrolysates

#### 2.5.1. Enzymolysis Analysis

The effects of various proteases (neutrase, acid protease, papain, flavourzyme, and alcalase) on the enzymatic hydrolysis of Flounder steaks were evaluated based on the contents of protein [19], amino acid nitrogen [22], and polypeptides [23] in enzymatic hydrolysates.

#### 2.5.2. Ferric Reducing Antioxidant Power (FRAP) Analysis

The total antioxidant capacity (T-AOC) of enzymatic hydrolysates was determined according to the potassium ferricyanide reduction method [24,25]. A total of 0.20 mL of enzymatic hydrolysate was mixed with 0.80 mL of purified water, 2.00 mL of phosphate buffer solution (0.20 moL/L, pH 6.6), and 2.00 mL of 1% K_3_FeCN_6_ solution. After heating at 50 °C for 20 min, 2.00 mL of 10% trichloroacetic acid (TCA) solution was added to terminate the reaction. The mixture was then centrifuged at 3000 rpm for 10 min, and 2.00 mL of the supernatant was mixed with 0.50 mL of 0.10% FeCl_3_ solution. The absorbance of the resulting mixture was measured at 700 nm. Glutathione (GSH) was used as the positive control.

#### 2.5.3. 2,2-Diphenyl-1-picrylhydrazyl (DPPH) Radical Scavenging Ability Analysis

The DPPH assay is widely recognized as a reliable method for evaluating free radical scavenging activity in ethanol-based systems [26]. In this study, 3.00 mL of 0.10 mmol/L DPPH solution prepared in absolute ethanol was added to 0.05 mL of enzymatic hydrolysate. The mixture was shaken and incubated for 30 min in the dark at room temperature. Following centrifugation at 10,000 rpm for 10 min, the absorbance of the supernatant was measured at 517 nm using a microplate reader. Distilled water and 95% ethanol served as the negative and positive controls, respectively. The DPPH radical scavenging activity (%) was calculated according to Equation (1):

DPPH radical scavenging activity (%) = 1 − (*A_x_* − *A_x_*_0_)/*A*_0_ × 100% (1)

where the *A_x_* is the absorbance of enzymatic hydrolysates; *A_x_*_0_ is the absorbance of positive control; and *A*_0_ is the absorbance of negative control.

#### 2.5.4. Emulsification (EAI) and Emulsifying Stability (ESI) Analysis

The EAI and ESI of enzymatic hydrolysates were determined according to the methods described in the references [27,28]. A total of 3.00 mL of enzymatic hydrolysates (10.00 mg/mL) at pH levels of 2, 4, 6, 8, and 12 were individually mixed with 1.00 mL of soybean oil. The mixtures were then stirred at 15,000 rpm for 1 min. Subsequently, 50.00 μL of the emulsified liquid collected from the bottom of each mixture was added to 5.00 mL of a 0.10% SDS solution. The absorbance values at 500 nm were measured immediately and after 10 min. The EAI (m^2^/g) and ESI (min) were calculated using Equations (2) and (3):

EAI (m^2^/g) = (2 × 2.303 × *A*_0_ × *N*)/(*L* × *C* × *Φ*) (2)

where *L* is 0.01 m; *N* is the dilution ratio of 100; and *C* is the protein concentration in the solution prior to emulsified liquid formation, g/m^3^. *Φ* is the volume fraction of oil in the emulsified liquid, %.

ESI (min) = (*A*_0_ × 10)/(*A*_0_ − *A*_10_) (3)

where *A*_0_ and *A*_10_ are the absorbance values of emulsified liquid at 500 nm before and after 10 min, respectively.

### 2.6. Optimization of MR Parameters

MR is the foremost approach for forming α-DCCs in seafood condiment production. The conditions of MR are the main factors influencing the concentrations of α-DCCs in the products [29,30]. Therefore, variations in temperature (ranging from 95 to 125 °C), time (10, 20, 30, 40, and 50 min), pH value (ranging from 5.0 to 9.0), and D-xylose concentration (1%, 2%, 3%, 4%, and 5%) were investigated in MR to optimize the processing technology and reduce the concentrations of 2,3-BD, MGO, GO, and 3-DG through reaction with 20.00 mg/mL of Flounder enzymatic hydrolysates.

### 2.7. Compound Flounder Seafood Condiments

After MR, the distinctive products were used to formulate Flounder seafood condiments, incorporating nucleotide disodium, citric acid, ginger powder, garlic powder, and yeast extract as batching agents. The effects of different ingredients on the α-DCC contents in the formulation of Flounder seafood condiments were evaluated based on the concentrations of 2,3-BD, MGO, GO, and 3-DG.

### 2.8. Quality Verification

Flounder seafood condiment samples were prepared according to the optimal formulation. Proximate composition analysis, sensory evaluation, and E-nose (isenso iNose, Shanghai, China) analysis were conducted to assess the quality characteristics. The results were then utilized to develop a feasible formula of Flounder seafood condiment with reduced α-DCC levels.

### 2.9. Statistical Analysis

Data management and analysis were performed by using SPSS 26.0 (IBM, SPSS Inc., Armonk, NY, USA). The experimental data collected in this study were expressed as means ± standard deviation (SD). Significant differences were identified using Duncan’s multiple range test at a 95% confidence level (*p*-value < 0.05). All graphs were drawn via GraphPad Prism 9.5 (San Diego, CA, USA).

## 3. Results and Discussion

### 3.1. Method Validation

A standard mixture of 2,3-BD, MGO, GO, and 3-DG was analyzed by LC-MS/MS to evaluate the linearity of different quantitation methods for four α-DCCs (Table 1 and Figure S1). The linear ranges of 2,3-BD, MGO, GO, and 3-DG were determined to be 0.05–10.00 ng/mL, 0.10–500.00 ng/mL, 5.00–1000.00 ng/mL, and 1.00–250.00 ng/mL, respectively. Calibration curves for these compounds exhibited excellent linearity within their respective concentration ranges (R^2^ > 0.99). The method detection limits (MDLs) at a signal-to-noise ratio of 3 (*S*/*N* = 3) were found to be 6.60, 5.30, 3.60, and 21.80 for 2,3-BD, MGO, GO, and 3-DG, respectively. Similarly, the lower limits of quantification (LOQs), determined at a signal-to-noise ratio of 10 (*S*/*N* = 10), were 10.70, 19.10, 10.50, and 24.00 for 2,3-BD, MGO, GO, and 3-DG, respectively. The sensitivity of the method was deemed satisfactory, which was feasible for accurately determining the concentrations of 2,3-BD, MGO, GO, and 3-DG.

### 3.2. Evaluation of the Proximate Composition in Flounder Steak

The proximate composition of Flounder steak is shown in Table 2. The moisture content in Flounder steak was 73.35 ± 0.44%, while the average contents of ash, lipid, and CHO were relatively lower at 5.18%, 3.13%, and 0.12%, respectively. Additionally, Flounder steak contained a higher protein content (approximately 16.96%), which was comparable to the protein content in Flounder meat (ranging from 17% to 19%) [31]. These results indicated that Flounder steak as a waste residue from processing still holds potential for further investigation and utilization as a protein source for seafood condiments.

### 3.3. Physicochemical Properties of Enzymatic Hydrolysates

#### 3.3.1. Evaluation of Enzymolysis

Enzymatic hydrolysis is a simple, safe, and environmentally friendly method currently used to convert fish by-products into more marketable and functional forms. Specifically, this process involves the hydrolysis of fish proteins [32]. Various proteases such as alcalase, flavourzyme, papain, neutrase, and acid protease have been used to prepare protein hydrolysates from fish by-products, which exhibit diverse biological activities [32,33].

**Figure 1:**
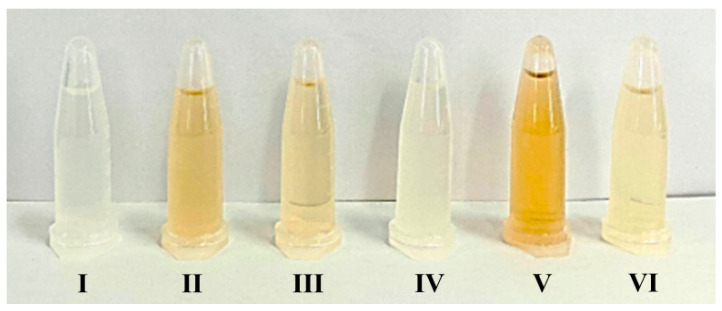
The enzymolysis liquids of Flounder steak. I. The Control, II. neutrase group, III. acid protease group, IV. papain group, V. flavourzyme group, and VI. alcalase group.

**Figure 2:**
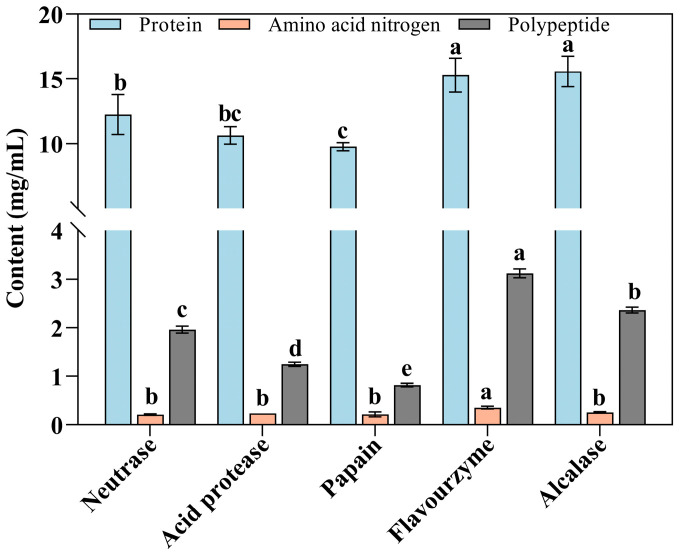
Effects of different proteases on protein, amino acid nitrogen, and polypeptide in enzymolysis liquids of Flounder steak (*n* = 3). Significant differences in the same index are indicated by different lowercase letters (a–e) (*p* < 0.05).

The various enzymolysis liquids of Flounder steak obtained using alcalase, flavourzyme, papain, neutrase, and acid protease are shown in Figure 1. Among them, the flavourzyme and alcalase groups appeared significantly effective for enzymatic hydrolysis (*p* < 0.05), with protein contents of 1.59 g/100 mL and 1.61 g/100 mL, respectively (Figure 2). In contrast, the papain group had a relatively lower protein content (0.98 g/100 mL) in the enzymatic hydrolysate. This may be attributed to the fact that hydrolysates derived from animal proteins are more effectively hydrolyzed by flavourzyme and alcalase, resulting in higher protein utilization rates [34]. Meanwhile, proteases gradually hydrolyzed the proteins in Flounder steaks into soluble proteins, peptides, and amino acids, which subtly contribute to the development of a distinctive flavor [35].

Amino acid nitrogen is a critical indicator of the degree of protein hydrolysis, with higher levels reflecting greater hydrolysis, more free amino groups, and better freshness [36]. Therefore, amino acid nitrogen content is commonly utilized as an indicator for assessing the quality of condiments such as soy sauce [37]. However, variations in enzymatic effects among different proteases are influenced by differences in their sites of action, specificity, and enzymatic activity, which may result in varying degrees of hydrolysis. In this study, the amino acid nitrogen content in the flavourzyme group was 36.05 mg/100 mL, significantly higher than that in the other groups (21.58 mg/100 mL, 23.45 mg/100 mL, 22.92 mg/100 mL, and 25.43 mg/100 mL in the neutrase, acid protease, papain, and alcalase groups, respectively) (*p* < 0.05). The results indicated that the flavourzyme could hydrolyze proteins in Flounder steak more thoroughly compared to other proteases (Figure 2).

The results of polypeptide contents in enzymolysis liquids were consistent with those of protein enzymolysis. In the flavourzyme group, the polypeptide content was 3.12 mg/mL, significantly higher than that in the other groups (*p* < 0.05). After enzymatic hydrolysis using alcalase, the polypeptide content in Flounder steaks was 2.36 mg/mL. These results indicated that flavourzyme and alcalase exhibit superior enzymatic hydrolysis effects on Flounder steak. Many studies have confirmed that the intake of natural high-efficiency antioxidant components could protect the body from free radical damage and regulate the REDOX balance [38]. Researchers favor food-derived protein peptides due to their high nutritional value, efficient absorption, and various biological activities, which showed vigorous antioxidant activity, such as oyster peptide [39] and cod peptide [40]. Therefore, higher polypeptide content could enhance the antioxidant capacity of enzymolysis liquids and inhibit the formation of carbonyl compounds during the MR.

#### 3.3.2. T-AOC and DPPH Radical Scavenging Ability Analysis

**Figure 3:**
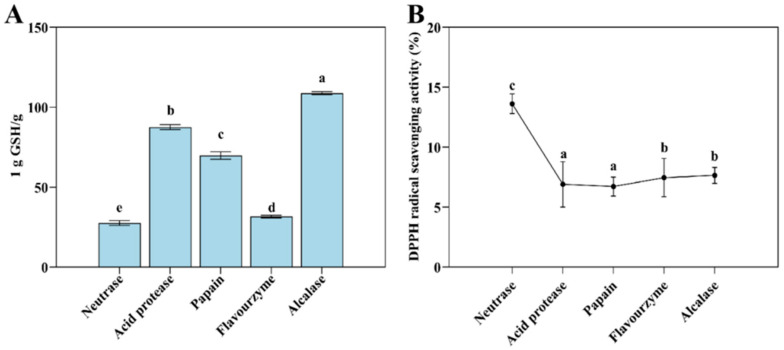
The T-AOC (**A**) and DPPH radical scavenging ability (**B**) of Flounder steak enzymatic hydrolysates (*n* = 3). Significant differences in the same index are indicated by different lowercase letters (a–e) (*p* < 0.05).

The hydrolysates of Flounder steak treated with different proteases showed varying levels of antioxidant activity, as evidenced by their T-AOC and DPPH radical scavenging capacity (Figure 3). Most enzymatic hydrolysates exhibited vigorous antioxidant activity after enzymolysis, while the alcalase group showed the lowest level of T-AOC (equivalent to 1/108.98 g GSH) and its DPPH radical scavenging capacity was 7.65% for 0.05 mL of the enzymatic hydrolysate. In contrast, the T-AOC of 0.20 mL neutrase enzymatic hydrolysate was equivalent to 1/49.41 g GSH, and its DPPH radical scavenging capacity increased to 13.62% for 0.05 mL of the enzymatic hydrolysate. In addition, the flavourzyme group showed higher DPPH radical scavenging ability (7.46%) and reducing power (equivalent to 1/31.50 g GSH). These results indicated that the T-AOC and DPPH radical scavenging ability of the neutrase and flavourzyme groups were superior to those of the other groups. This is likely due to neutrase’s ability to hydrolyze hydrophobic proteins and expose more hydrophobic groups, thereby enhancing antioxidant activity. Flavourzyme, which functions as both an endopeptidase and exopeptidase, effectively hydrolyzes hydrophobic peptides, potentially contributing to its enhanced antioxidant capacity in alcohol solutions.

Nowadays, fish by-products, which are often underutilized, represent a valuable resource for extracting bioactive peptides with antioxidant properties [41]. An increasing number of studies have demonstrated the utilization of fish by-products for preparing antioxidant peptide fractions, such as those derived from rainbow trout (*Onchorhynchus mykiss*) viscera [42], tuna (*Thunnus albacares*) [43], and Atlantic cod (*Gadus morhua*) processing by-products [44]. This is because enzymatic hydrolysis can expose more free amino termini, which show specific reducing actions due to their ability to scavenge free radicals. In addition, the reducibility of these hydrolysates can limit the MR by slowing down the reaction rate and contribute to reducing α-DCCs in food [45].

#### 3.3.3. Evaluation of the EAI and ESI

**Figure 4:**
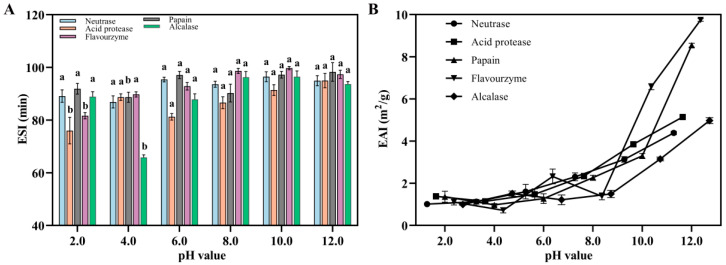
The ESI (**A**) and EAI (**B**) of various Flounder steak enzymatic hydrolysates. There were significant differences in the same index by different lowercase letters (a and b) (*p* < 0.05).

EAI represents the ability of polypeptides to form an emulsion in a water–oil mixture, while ESI reflects the ability of the emulsion to remain stable under external pressure. Several factors, such as strength, temperature, and oil phase volume, influence the emulsifying properties of proteins to some extent, but the EAI of proteins primarily depends on their solubility. In this study, Flounder steak was enzymatically hydrolyzed using different proteases, which increased the solubility of polypeptides owing to the exposure of hydrophobic residues. These hydrophobic residues enhanced the binding ability between polypeptides and oil phase, resulting in a noticeable EAI effect. In addition, the EAI of enzymatic hydrolysates increased with rising pH values, and the ESI showed a rapid growth trend when the pH value exceeded 8 (Figure 4). In the flavourzyme group, the ESI at different pH values consistently exceed 80 min and the highest EAI reached more than 9.00 m^2^/g, making it suitable for product development. The results demonstrated that, after enzymolysis, the enzymatic hydrolysates of Flounder steak exhibited preferable EAI and ESI in alkaline solution, particularly those in the flavourzyme group. These results indicated that, when the pH value exceeded 7.0, the EAI and ESI were higher than those under acidic conditions. This is because, under different pH conditions, the EAI is affected due to the effect of electric charge. As the pH increases at the oil–water interface, the negative charge on the protein surface increases, which enhances the repulsion and increases the thickness of the protein hydration layer, thus improving the emulsification stability [46]. Meanwhile, better emulsifying activities suggest hydrolysates with low molecular weights and superior solubility, which offer advantages in food processing applications [47]. Therefore, the enzymatic hydrolysates of Flounder steak in the flavourzyme group are more suitable for the subsequent research.

### 3.4. Effects of MR Parameters on α-DCC Contents

**Figure 5:**
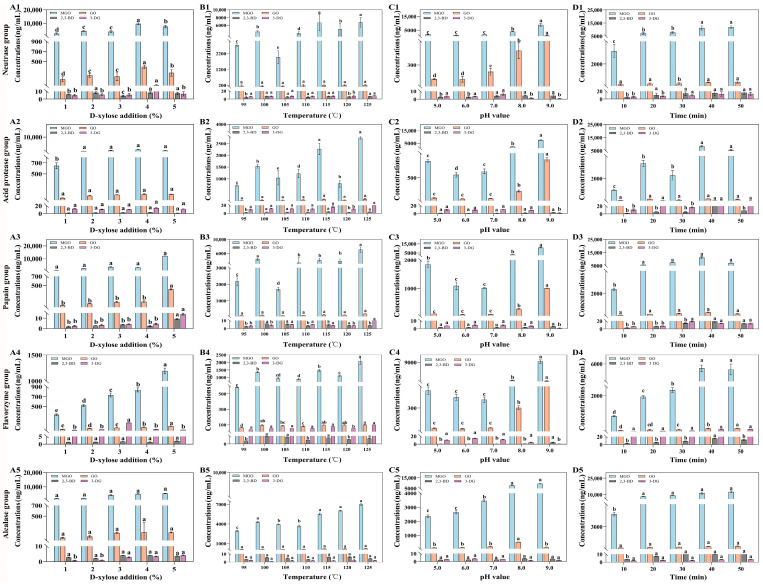
The effects of D-xylose addition (**A1**–**A5**), temperature (**B1**–**B5**), pH value (**C1**–**C5**), and reaction time (**D1**–**D5**) on the concentrations of MGO, GO, 2,3-BD, and 3-DG in MR. Significant differences in the same index are indicated by different lowercase letters (a–d) (*p* < 0.05).

The effects of various MR parameters on the contents of MGO, GO, 2,3-BD, and 3-DG are shown in Figure 5. D-xylose is a common reducing sugar used in thermal food processing to facilitate the MR. D-xylose at concentrations of 1%, 2%, 3%, 4%, and 5% was added to 50.00 mL of 20.00 mg/mL Flounder steak enzymolysis liquids (pH 8.5), and the mixture was reacted at 105 °C for 25 min. With the concentrations of D-xylose increased from 1% to 4%, the degree of MR became more intense, which caused an increase in the total concentrations of MGO, GO, 2,3-BD, and 3-DG in all groups (Figure 5(A1–A5)). When the concentration of D-xylose reached 5%, the total concentrations of four α-DCCs continued to increase in the papain, flavourzyme, and acid protease groups (from 3432.70 μg/mL, 946.95 μg/mL, and 5295.82 μg/mL to 12,640.93 μg/mL, 1328.53 μg/mL, and 5606.79 μg/mL, respectively), primarily due to the increasing formation of MGO through MR. In contrast, the contents of four α-DCCs in the alcalase and acid protease groups decreased compared to those at a D-xylose concentration of 4%. In addition, after MR, the total concentration of MGO, GO, 2,3-BD, and 3-DG in the flavourzyme group was the lowest. The amounts of 2,3-BD and 3-DG increased less distinctly compared to those of MGO and GO. So, the concentration of D-xylose in MR should be kept below 4%. Meanwhile, the reducing capacity of the enzymatic hydrolysates might contribute to decreasing the concentrations of α-DCCs.

Temperature is a main factor influencing the extent of MR by changing the degrees of molecular motion and dissociation. As the temperature of MR increased, the amount of reaction products increased significantly due to the faster reaction rate [48]. As shown in Figure 5(B1–B5), the concentrations of α-DCCs were lowest at 95 °C. When the reaction temperature of MR (3% D-xylose pH 8.5 for 30 min) exceeded 110 °C, the total concentrations of four α-DCCs increased dramatically, which might be related to the increasing content of MGO. Among different groups, the concentrations of GO were all less than 230.00 ng/mL, whereas the concentration of MGO was more than 7000.00 ng/mL in the papain group. The minimum total contents of MGO, GO, 2,3-BD, and 3-DG were 2249.29 ng/mL in the flavourzyme group. Therefore, considering both flavor factors and α-DCCs concentrations, 105 °C should serve as the reference temperature for MR in the preparation of seafood seasoning.

Figure 5(C1–C5) shows the effects of pH variations on the concentrations of MGO, GO, 2,3-BD, and 3-DG. The concentrations of four α-DCCs at pH 8.0 were more than twice as high as those at pH 7.0. When the pH value reached 9.0, the increasing rates of MGO and GO became more evident, leading to total concentrations exceeding 8000.00 ng/mL, although the contents of 2,3-BD and 3-DG decreased. This is because hydrogen or hydroxide ions in the reaction solution either inhibit or promote the dissociation of carboxyl and amino groups in the system, thereby suppressing or enhancing the reaction. In acidic conditions, the presence of the amino group -NH_3_^+^ hinders the condensation of the carbonyl group, resulting in less pronounced browning. Under alkaline conditions, the amino acid nitrogen is released from the reaction, and the higher the alkalinity, the stronger the reaction [49]. Hence, the suitable pH range for MR should be set between 6.0 and 7.0.

Figure 5(D1–D5) showed the effects of different reaction times on the concentrations of MGO, GO, 2,3-BD, and 3-DG in MR using 50.00 mL of 20.00 mg/mL Flounder steak enzymatic hydrolysates (4% D-xylose, pH 6.5) at 105 °C. The experimental results indicated that the concentrations of the four α-DCCs significantly increased with increasing heating time. This is probably because the reaction was terminated at 40 min, at which point the degree of browning had reached its maximum. As the reaction time was extended, substantial water evaporation from the system resulted in higher product concentrations. Furthermore, studies have indicated that the nutrient levels in fish decrease over time [50]. Therefore, the reaction time of MR should be controlled between 10 min and 30 min.

### 3.5. Analysis of α-DCCs in Commercially Available Seafood Condiments

**Figure 6:**
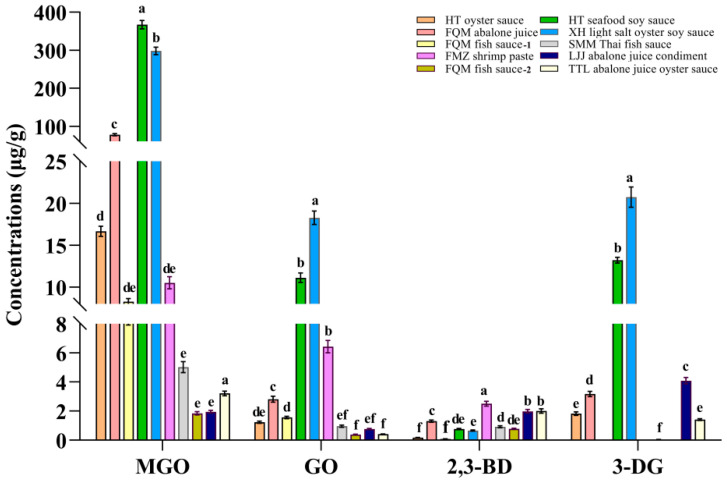
Quantification of MGO, GO, 2,3-BD, and 3-DG in 10 commonly available seafood condiments on the market (*n* = 3). Significant differences in the same index are indicated by different lowercase letters (a–f) (*p* < 0.05).

Ten commercially available seafood condiments were selected, representing five product categories: soy sauce, oyster sauce, abalone sauce, fish sauce, and shrimp paste. The results showed that the highest levels of MGO, GO, and 3-DG were detected in soy sauce, while the lowest levels were observed in fish sauce (Figure 6). The concentrations of MGO in the 10 condiments ranged from 1.84 to 367.00 μg/g and were significantly higher in seafood soy sauce compared to other groups (*p* < 0.05). The concentrations of GO were 0.39–18.27 μg/g, with the highest concentration found in oyster sauce (*p* < 0.05). The concentrations of 2,3-BD and 3-DG ranged from 0.09 to 2.50 μg/g and 0 to 20.74 μg/g, respectively (Table S1). A previous analytical study on the content of three α-DCCs in commercial soy sauce in Korea revealed that, among 20 soy sauce samples, all but one contained the highest amount of MGO, followed by GO and 2,3-BD [51], which was similar with the experimental results of this research. As consumer awareness of food safety has increased, numerous studies have highlighted the potential adverse effects of MR products on human health. For instance, the US maximum daily intake level for MGO is 3.00 μg/d and the EU standard maximum daily intake level for 2,3-BD is 2200.00 μg/d [52]. In addition, the cytotoxicity threshold for GO is 0.25 mM and the recommended daily intake range for 3-DG is 20.00–160.00 mg/d [53]. Therefore, reducing the contents of MGO, GO, 2,3-BD, and 3-DG in seafood condiments could effectively lower the daily intake of α-DCCs.

### 3.6. Effects of Ingredients on α-DCC Contents

**Figure 7:**
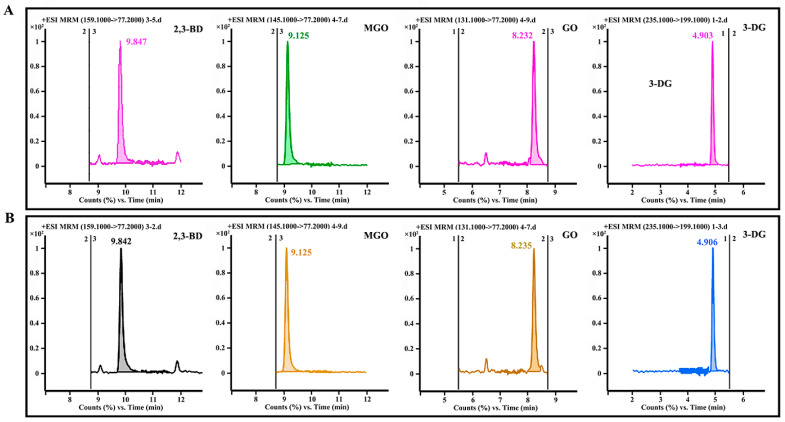
LC-MS/MS chromatograms of MGO, GO, 2,3-BD, and 3-DG in seafood condiments prepared using enzymolysis liquids: (**A**) flavourzyme group and (**B**) papain group.

The contents of α-DCCs in seafood condiments were related to the differences in production techniques [17]. MR conditions were the main factors influencing the levels of α-DCCs in seafood condiments. According to the results of MR, the concentrations of four α-DCCs were lowest in the flavourzyme group owing to its high polypeptide content and antioxidant capacity. Consequently, an optimized formulation (A1B1C1D2) derived from the flavourzyme group was applied to prepare Flounder seafood condiments by using 50.00 mL of MR products and 55.00 g of gelatinized starch, 2.25 g of salt, 0.10 g of nucleotide disodium, 0.10 g of citric acid, 0.05 g of ginger powder, 0.10 g of garlic powder, and 0.10 g of yeast extract as the batching (Table S2). The optimized formulation (A1B3C1D1) obtained from the papain group served as the control (Table S3). The concentrations of MGO, GO, 2,3-BD, and 3-DG determined by LC-MS/MS were reduced to 1.23 ± 0.02, 0.23 ± 0.01, 0.01 ± 0.12 × 10^−2^, and 0.05 ± 0.19 × 10^−2^ μg/g in the flavourzyme group and 1.80 ± 0.17, 0.27 ± 0.01, 0.01 ± 0.16 × 10^−2^, and 0.08 ± 1.03 × 10^−2^ μg/g in the papain group, respectively (Figure 7), which showed that the ingredients could not significantly increase the contents of four α-DCCs.

α-DCCs are the vital intermediates in MR. They are formed endogenously during food processing. Currently, comprehensive data on α-DCCs showed that the highest total dicarbonyl concentrations were found in dried fruit, Dutch spiced cake, and candy bars (>400 mg/kg), while the lowest concentrations occurred in tea, dairy, light soft drinks, and rice (<10 mg/kg) [54]. However, there were fewer data about the contents of α-DCCs in aquatic products and their derivatives. The aim of this study was to analyze and reduce four α-DCCs via optimizing MR parameters during the processing of Flounder condiment. Compared with 10 commercially available seafood condiments, the total content of four α-DCCs in the prepared Flounder condiment was relatively lower (<5 mg/kg), indicating that the contents of four α-DCCs in the prepared Flounder condiment comply with the standards set by the European Union and the United States.

### 3.7. Optimization Evaluation of Flounder Seafood Condiment Formulation

The Flounder seafood condiment sample from the flavourzyme group was selected as the optimal formulation for quality verification according to the contents of four α-DCCs. The Flounder seafood condiment was semi-liquid, with a water content of 78.82 ± 0.08%. Additionally, the ash, crude protein, crude fat, and total sugar of the Flounder condiment were 2.05 ± 0.29%, 6.31 ± 0.20%, 3.06 ± 0.31%, and 4.72 ± 0.25%, respectively.

The sensory scores initially increased and then decreased with the addition of gelatinized starch, salt, and MR products (Table 3, Tables S4 and S5). The highest score was achieved at 55.00 g of gelatinized starch. As the addition of gelatinized starch increased, the condiment became stickier and the fish flavor diminished. A salt level of 2.25 g effectively suppressed bitterness while enhancing the umami taste of the condiment. Adding 50.00 mL of the MR solution could increase the freshness of the seasoned fish; however, exceeding this volume resulted in a diluted and less appetizing condiment. Finally, the optimal Flounder seafood condiment sample scored 89.79, which was higher than that of other samples.

**Figure 8:**
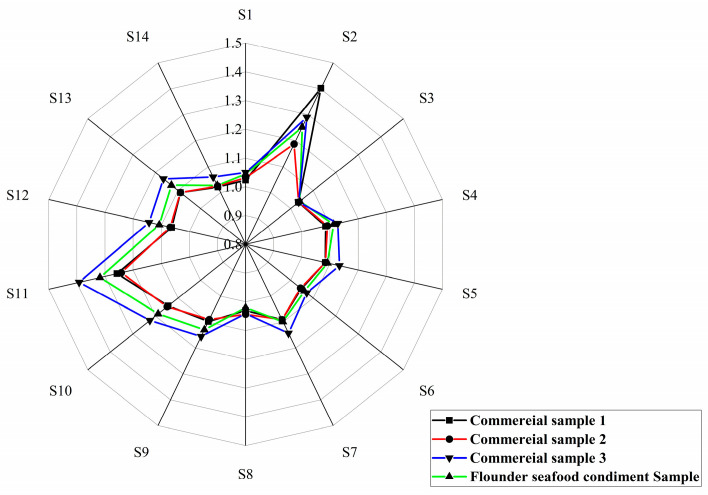
The E-nose analysis of Flounder seafood condiment with MR product from the flavourzyme group.

The electronic nose data showed that the optimal Flounder seafood condiment exhibited a similar odor profile to the commercial products, with an overall odor intensity higher than that of the market products. The intensity of sulfides in Flounder seafood condiment (Figure 8 S2 sensor) was intermediate in flavor, originating from the breakdown of sulfur-containing amino acids and contributing a certain spoilage odor to the product. The Flounder seafood condiment sample demonstrated significantly higher intensities for alkanes, alcohols (Figure 8 S11 sensor), and cooking smells (Figure 8 S13 sensor) compared to market products. These compounds were primarily the final products of the MR and high-temperature decomposer of organic matter. Amines, which are commonly associated with microbial spoilage and volatile organic compounds derived from fossil fuels (Figure 8 S1 sensor), showed lower levels in the Flounder seafood condiment. The results demonstrated that the optimal Flounder seafood condiment formulation, incorporating MR product from the flavourzyme group, possessed excellent quality and was suitable for the preparation of commercial Flounder seafood condiments.

Currently, the primary raw materials for producing of seafood condiments typically include low-value miscellaneous fish, shrimp, and aquatic processing by-products (e.g., skin, bone, head, and tail), as well as secondary products such as cooking juices and cooking liquids. These materials not only contain abundant flavor substances but are also rich in nutrients. Through the MR and ingredient addition, the prepared seafood condiments exhibit a diverse range of aroma components, including aldehydes, alcohols, furs, ketones, nitrogen compounds, sulfur compounds, esters, hydrocarbons, and phenols. During the cooking process, the application of seafood condiments can compensate for the loss of characteristic aroma caused by cooking and address the issue of high aquatic product consumption. Although they cannot fully replace the seafood ingredients, seafood condiments enhance the taste and aroma of products to some extent and help maintain their sensory qualities, thereby satisfying the diverse needs of consumers [55].

The MR can effectively enhance the flavor of aquatic products and improve the functional properties of aquatic proteins, demonstrating significant application potential in seafood condiment production. However, the MR is complex, producing a wide variety of reaction products, some of which may include harmful substances such as α-DCCs and advanced glycosylation end products (AGEs). According to the findings of this paper, we determined that α-DCCs can be reduced and controlled by optimizing the parameters of MR. The prepared Flounder seafood condiment showed product characteristics similar to those of commercially available seafood condiments. Therefore, the reaction process should be reasonably controlled to guide the reaction toward the desired direction while minimizing the formation of α-DCCs and other harmful substances. With further research into the mechanism of MR, its applications in the aquatic processing industry will become even more extensive. Developing rapid detection methods for α-DCCs during the MR in aquatic product processing represents a valuable area for further investigation.

## 4. Conclusions

This study developed a new seafood condiment with reduced α-DCC contents using Flounder steak as the raw material. The parameters of MR, including temperature, reaction time, pH value, and D-xylose addition, were optimized to minimize the concentrations of MGO, GO, 2,3-BD, and 3-DG in MR products prepared from enzymolysis liquids of alcalase, flavourzyme, papain, neutrase, and acid protease, respectively. Due to the lower α-DCC contents in the MR product, an optimized formulation from the flavourzyme group was applied to prepare the Flounder seafood condiment. In this formulation, MGO, GO, 2,3-BD, and 3-DG were reduced to 1.23 ± 0.02, 0.23 ± 0.01, 0.01 ± 0.12 × 10^−2^, and 0.05 ± 0.19 × 10^−2^ μg/g, respectively, and scored 89.79 in sensory evaluation. The concentrations of 2,3-BD, MGO, GO, and 3-DG were lower than the lowest levels detected in 10 commercial seafood condiments via LC-MS/MS, conforming to the daily intake standards set by the United States and the European Union. The development of this new Flounder-based seafood condiment provides an effective approach for utilizing aquatic products and increasing the market value of Flounder by-products. More importantly, it raises the possibility of reducing dicarbonyl compounds in seafood condiments, which may have adverse physiological effects on human health. However, the optimization of MR parameters is complex, and the detection of α-DCCs requires laboratory-based LC-MS/MS analysis, which might limit further study into reducing α-DCCs in the field of aquatic product processing.

## Figures and Tables

**Table 1:** Linear ranges, regression equations, MDLs, and LOQs of four α-DCCs.

Analyte	Regression Equation	Correlation Coefficient	Linear Range (ng/mL)	MDL(μg/kg)	LOQ (μg/kg)
2,3-BD	y = 685.78x + 330.04	0.991	0.05–10.00	6.60	10.70
MGO	y = 685.78x + 330.04	0.999	0.10–500.00	5.30	19.10
GO	y = 13.526x	0.999	5.00–1000.00	3.60	10.50
3-DG	y = 31,615x + 302.12	0.991	1.00–250.00	21.80	24.00

**Table 2:** The proximate components of Flounder steak.

Sample	Moisture/%	Protein/%	Ash/%	Lipid/%	CHO/%
Flounder steak	73.35 ± 0.44	16.96 ± 0.39	5.18 ± 0.22	3.13 ± 0.16	0.12 ± 0.05

**Table 3:** The sensory scores of optimal Flounder seafood condiment prepared using MR product from the flavourzyme group.

No.	Gelatinized Starch Addition/g	Salt/g	MR Product/mL	Sensory Scores
1	65	2.25	45	80.23
2	55	2.00	55	83.16
3	65	1.75	55	75.23
4	55	2.25	50	87.70
5	60	2.25	55	81.96
6	65	2.00	50	79.10
7	60	2.00	45	81.06
8	60	1.75	50	76.03
9	55	1.75	45	75.23

## Data Availability

The original contributions presented in the study are included in the article/Supplementary Material, further inquiries can be directed to the corresponding author.

## Supplementary Materials

The following supporting information can be downloaded at: https://www.mdpi.com/article/10.3390/foods14101717/s1, Figure S1. Four α-dicarbonyl compounds’ chromatographic information. Figure S2. The chromatograms of 10 commercially available seafood condiments. Table S1. The concentrations of MGO, GO, 2,3-BD, and 3-DG in 10 commonly available seafood condiments on the market. Table S2. The optimization of MR process parameters for Flounder condiment formulation in the flavourzyme group. Table S3. The optimization of MR process parameters for Flounder condiment formulation in the papain group. Table S4. Sensory evaluation indicators for Flounder seafood condiment. Table S5. Sensory scoring criteria for Flounder seafood condiment.
