# Supplementary material for: Reduction and Control Technology of Harmful Dicarbonyl Compounds in Flounder (Pleuronectiformes) Seafood Condiment Preparation
Source: Foods. 2025 May 12;14(10):1717. doi: 10.3390/foods14101717 (PMC12110771; doi:10.3390/foods14101717)
Supplement: Supplementary file 1 [file foods-14-01717-s001.zip › foods-3521391-supplementary.pdf]

Supplementary Data

A-1

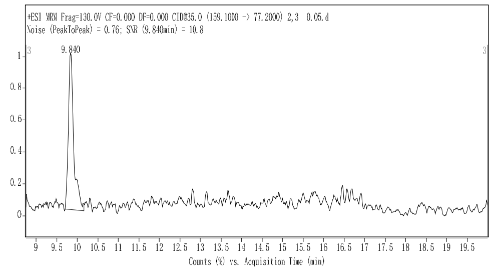

A-2

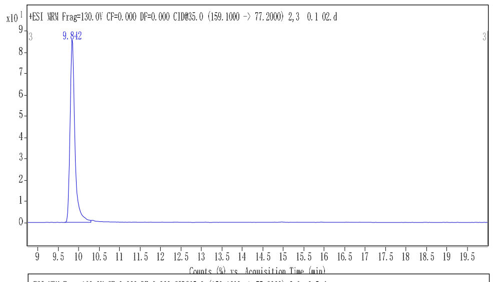

A-3

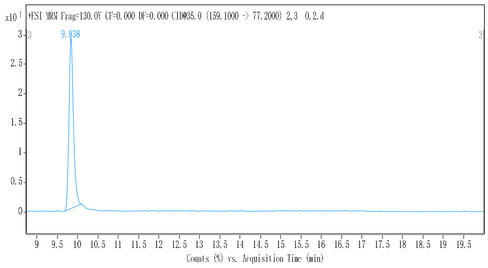

A-4

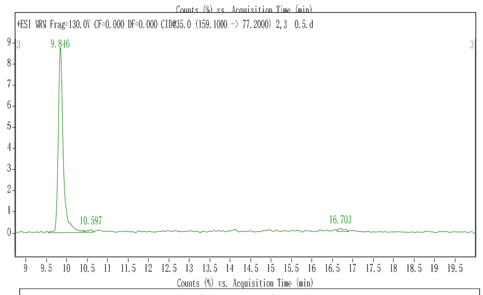

A-5

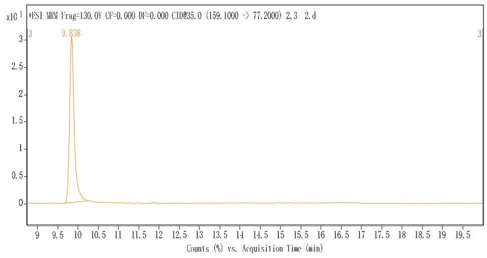

A-6

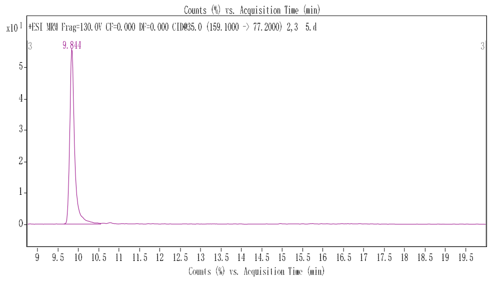

A-7

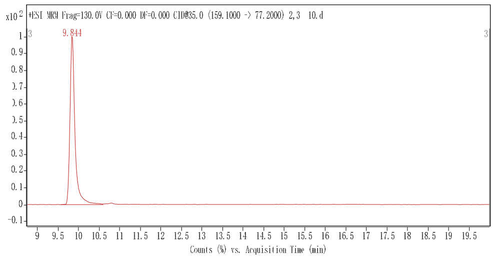

B-1

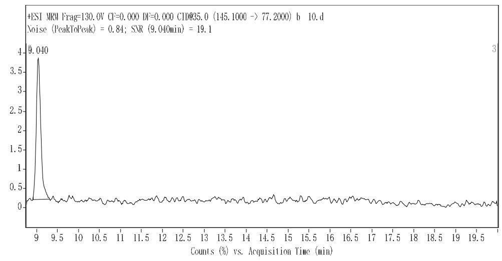

B-2

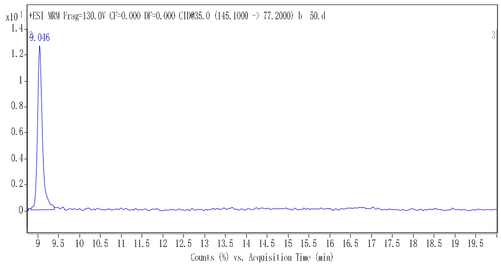

B-3

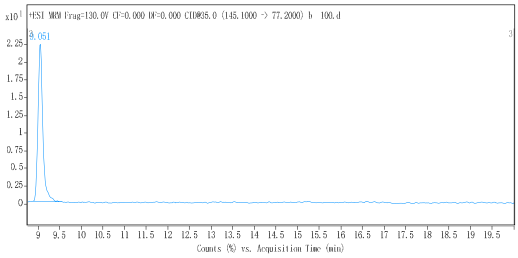

B-4

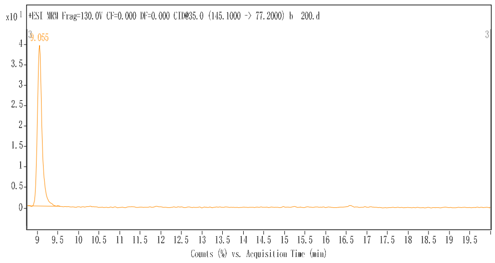

B-5

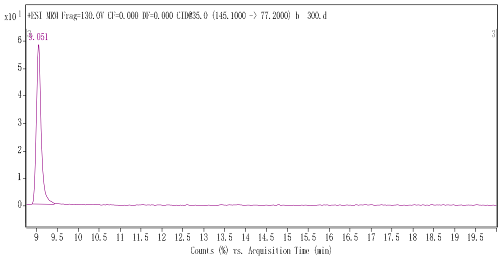

B-6

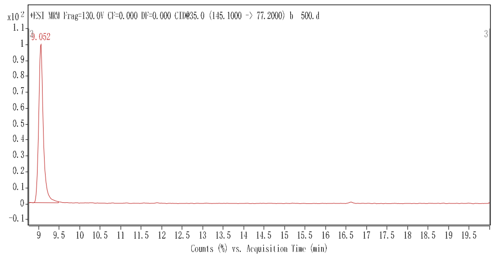

C-1

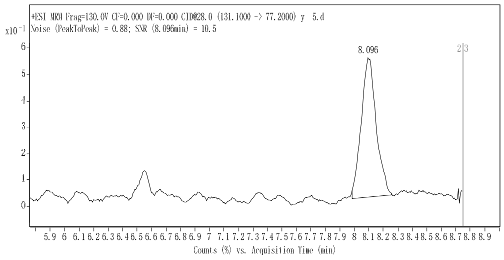

C-2

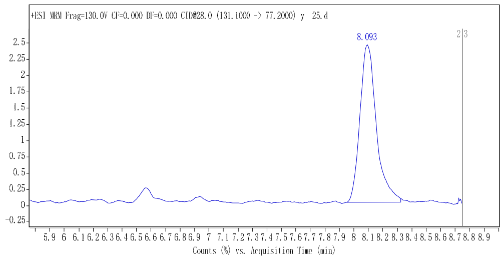

C-3

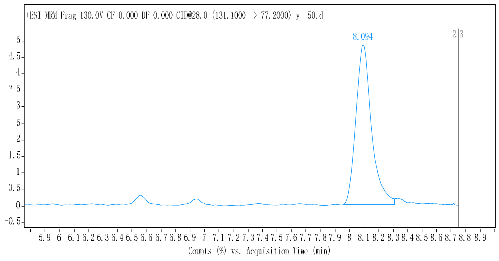

C-4

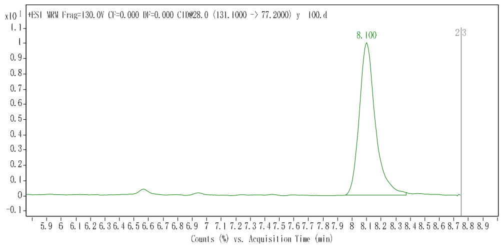

C-5

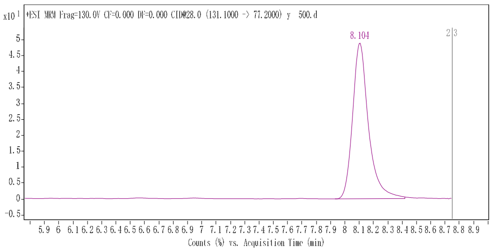

C-6

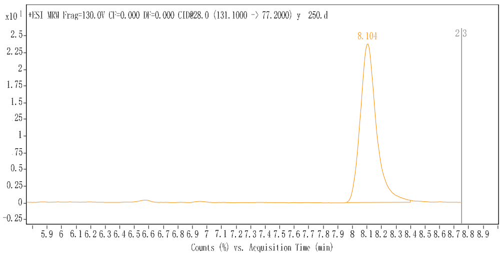

C-7

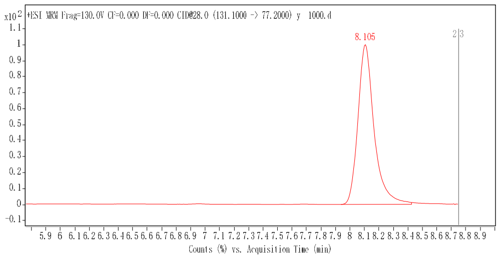

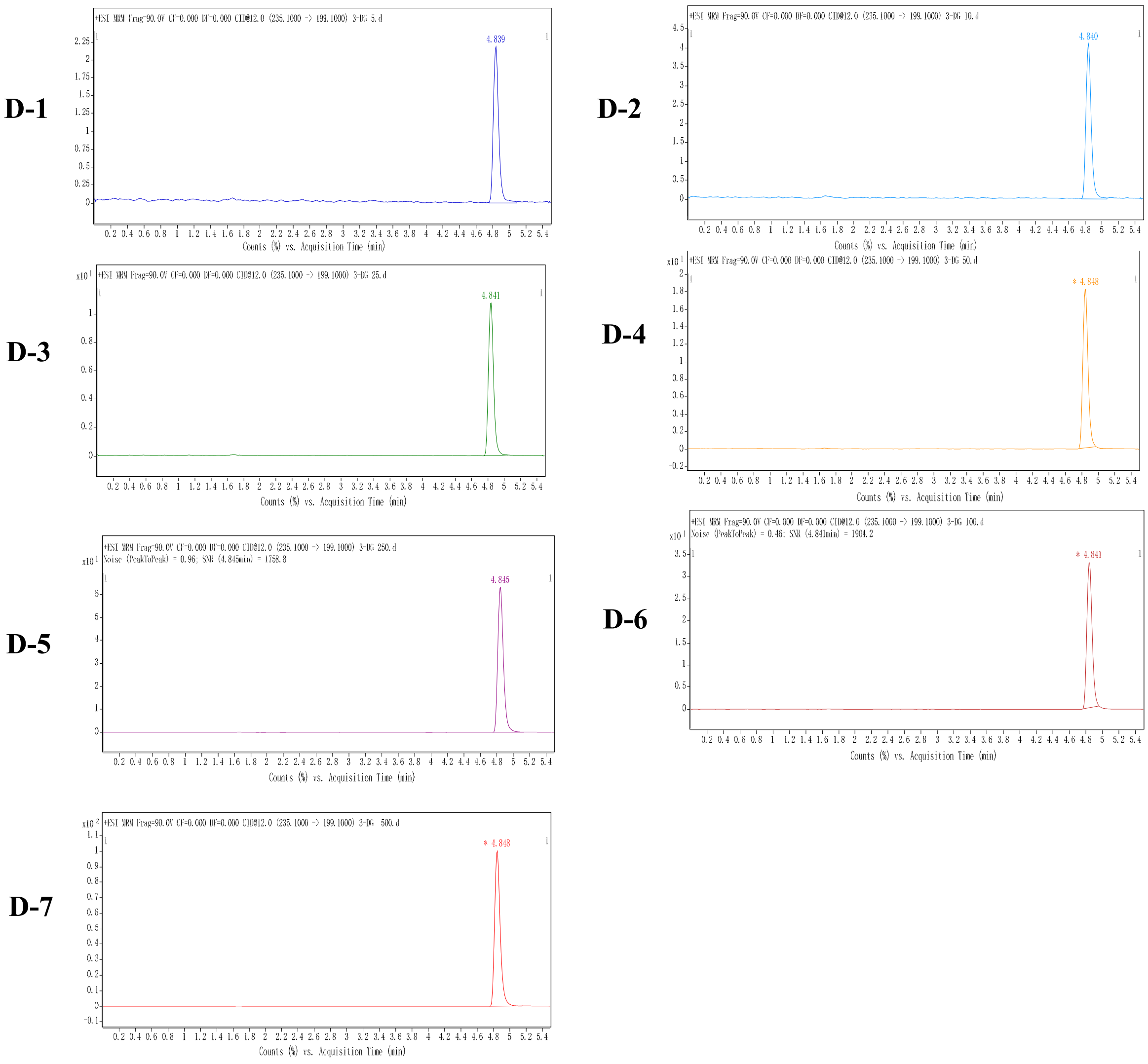

Figure S1. Four α-dicarbonyl compounds chromatographic information.

(**A-1**: 0.05 ng/mL of 2,3-BD; **A-2**: 0.10 ng/mL of 2,3-BD; **A-3**: 0.20 ng/mL of 2,3-BD; **A-4**: 0.50 ng/mL of 2,3-BD; **A-5**: 2.00 ng/mL of 2,3-BD; **A-6**: 5.00 ng/mL of 2,3-BD; **A-7**: 10.00 ng/mL of 2,3-BD; **B-1**: 10.00 ng/mL of MGO; **B-2**: 50.00 ng/mL of MGO; **B-3**: 100.00 ng/mL of MGO; **B-4**: 200.00 ng/mL of MGO; **B-5**: 300.00 ng/mL of MGO**; B-6**: 500.00 ng/mL of MGO; **C-1**: 5.00 ng/mL of GO; **C-2**: 25.00 ng/mL of GO; **C-3**: 50.00 ng/mL of GO; **C-4**: 100.00 ng/mL of GO; **C-5**: 250.00 ng/mL of GO; **C-6**: 500.00 ng/mL of GO; **C-7**: 1000.00 ng/mL of GO; **D-1**: 1.00 ng/mL of 3-DG; **D-2**: 5.00 ng/mL of 3-DG; **D-3**: 10.00 ng/mL of 3-DG; **D-4**: 25.00 ng/mL of 3-DG; **D-5**: 50.00 ng/mL of 3-DG; **D-6**: 100.00 ng/mL of 3-DG; **D-7**: 250.00 ng/mL of 3-DG; **D-8**: 500.00 ng/mL of 3-DG.)

Table S1. The concentrations of MGO, GO, 2,3-BD, and 3-DG in 10 commonly available seafood condiments on the market.

10 seafood condiments samples	Concentrations of α-DCCs (μg/g)			
	MGO	GO	2,3-BD	3-DG
HT oyster sauce	16.65 ± 0.54	1.23 ± 0.07	0.17 ± 0.02	1.83 ± 0.10
FQM abalone juice	77.69 ± 2.51	2.82 ± 0.18	1.31 ± 0.06	3.17 ± 0.17
FQM fish sauce-1	8.27 ± 0.32	1.56 ± 0.08	0.09 ± 0.01	nd
HT seafood soy sauce	367.13 ± 9.90	11.12 ± 0.51	0.77 ± 0.04	13.22 ± 0.30
XH light salt oyster soy sauce	298.34 ± 8.90	18.27 ± 0.77	0.67 ± 0.03	20.74 ± 1.08
FMZ shrimp paste	10.52 ± 0.65	9.43 ± 0.38	2.50 ± 0.15	nd
SMM Thai fish sauce	5.02 ± 0.34	0.96 ± 0.07	0.92 ± 0.06	0.05 ± 0.01
FQM fish sauce-2	1.84 ± 0.10	0.39 ± 0.02	0.79 ± 0.04	nd
LJJ abalone juice condiment	1.95 ± 0.09	0.77 ± 0.04	1.98 ± 0.12	4.09 ± 0.20
TTL abalone juice oyster sauce	3.21 ± 0.14	0.41 ± 0.02	2.00 ± 0.13	1.42 ± 0.05

A

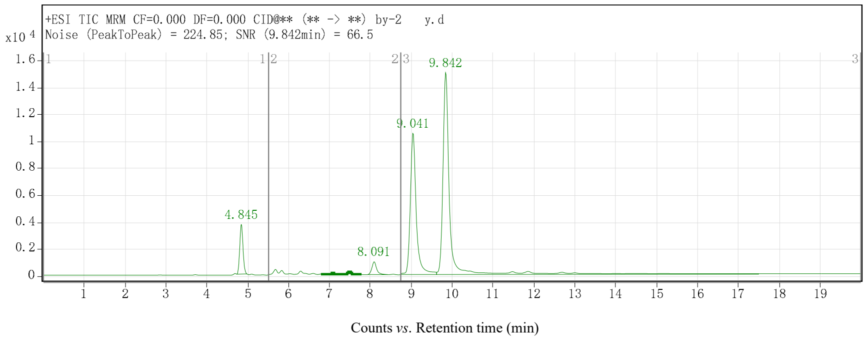
+ESI TIC MRM CF=0.000 DF=0.000 CID@** (** -> **) by-2 y.d
Noise (PeakToPeak) = 224.85; SNR (9.842min) = 66.5
x10 4
4.845
8.091
9.041
9.842
Counts vs. Retention time (min)

B

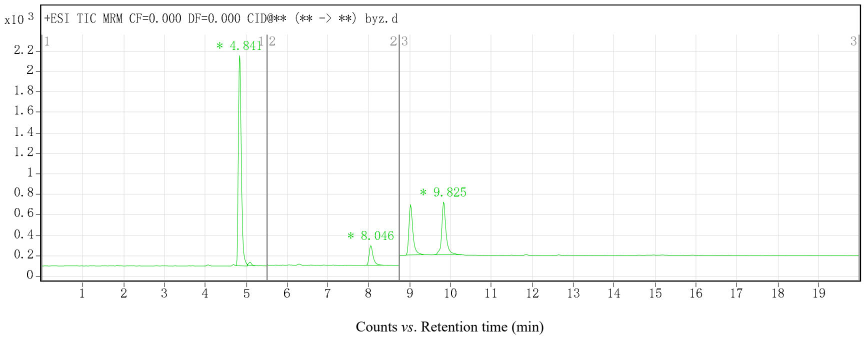
+ESI TIC MRM CF=0.000 DF=0.000 CID@** (** -> **) byz.d
x10 3
* 4.841
* 8.046
* 9.825
Counts vs. Retention time (min)

C

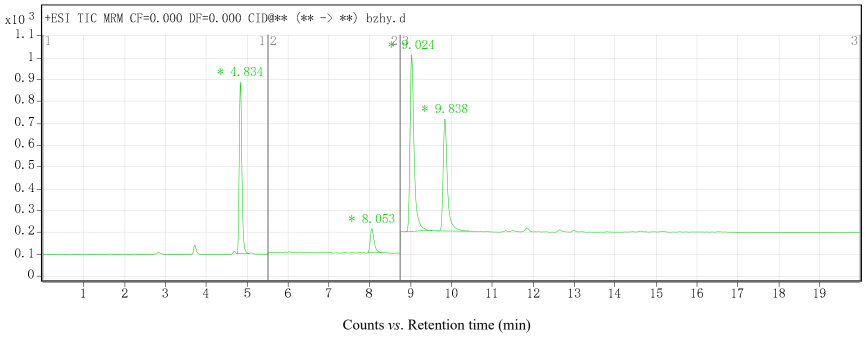
+ESI TIC MRM CF=0.000 DF=0.000 CID@** (** -> **) bzhy.d
x10 3
* 4.834
* 8.053
* 9.024
* 9.838
Counts vs. Retention time (min)

D

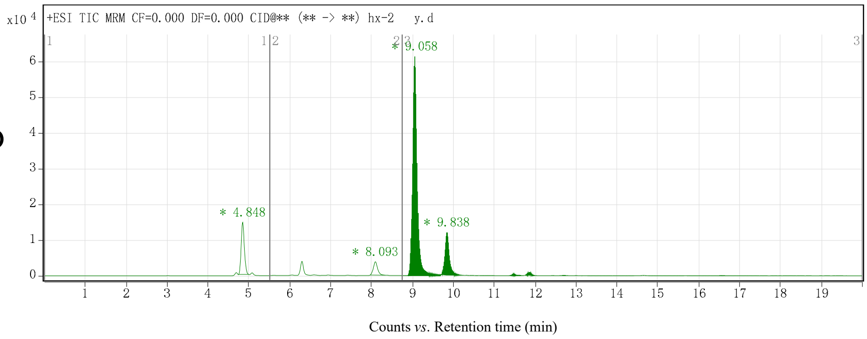
+ESI TIC MRM CF=0.000 DF=0.000 CID@** (** -> **) hx-2 y.d
x10 4
* 4.848
* 8.093
* 9.058
* 9.838
Counts vs. Retention time (min)

E

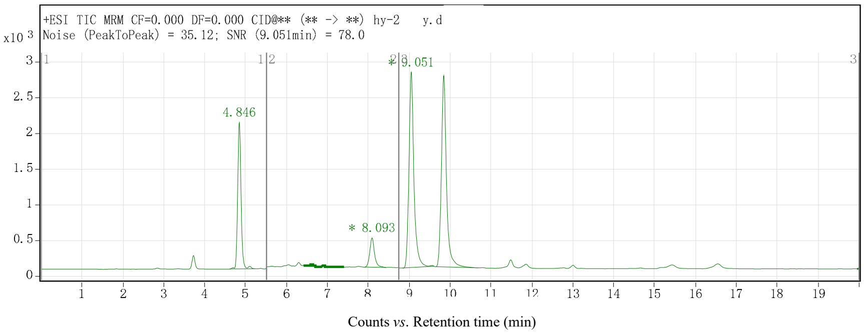
+ESI TIC MRM CF=0.000 DF=0.000 CID@** (** -> **) hy-2 y.d
Noise (PeakToPeak) = 35.12; SNR (9.051min) = 78.0
x10 3
4.846
* 8.093
* 9.051
Counts vs. Retention time (min)

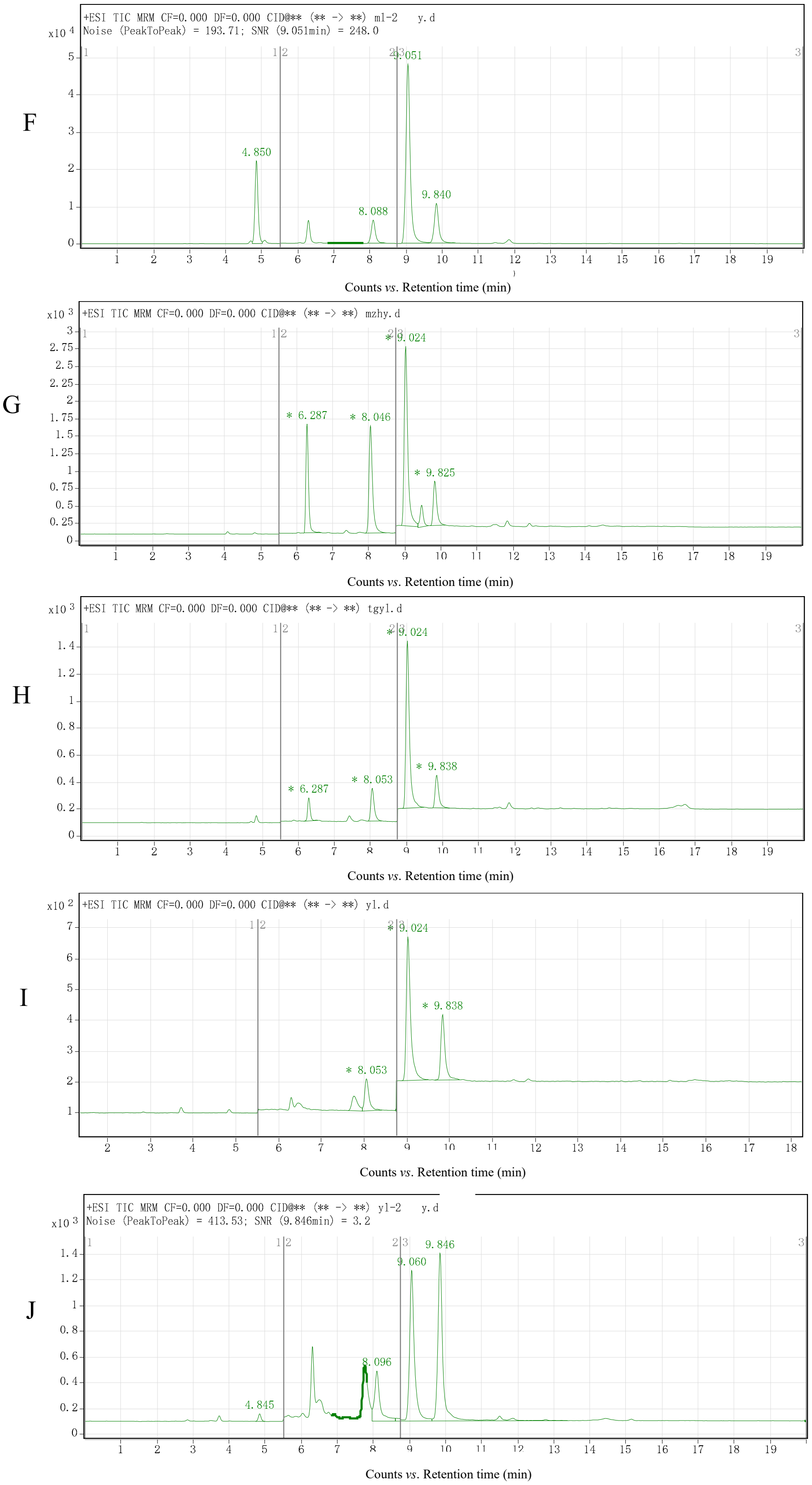

Figure S2. The chromatograms of 10 commercially available seafood condiments.

A. FQM abalone juice, **B.** LJJ abalone juice condiment, **C.** TTL abalone juice oyster sauce, **D.** HT seafood soy sauce, **E.** HT oyster sauce, **F.** XH light salt oyster soy sauce, **G.** FMZ shrimp paste, **H.** SMM Thai fish sauce, **I.** FQM fish sauce-1, **J.** FQM fish sauce-1.

Table S2. The optimization of MR process parameters for Flounder condiment formulation in the flavourzyme group.

No.		D-xylose addition (%) (A)	pH value (B)	Temperature (℃) (C)	Time (min) (D)	MGO (ng/mL)	GO (ng/mL)	3-DG (ng/mL)	2,3-BD (ng/mL)
1		2.5	6.5 (1)	110.0 (3)	20.0 (2)	348.38 ± 11.27	43.65 ± 1.21	2.54 ± 0.13	1.35 ± 0.06
2		1.5 (1)	7.0 (2)	110.0	25.0 (3)	330.29 ± 8.21	45.38 ± 1.70	3.23 ± 0.11	1.59 ± 0.12
3		2.5 (3)	7.0	107.5 (2)	15.0 (1)	283.01 ± 3.38	37.05 ± 1.24	2.46 ± 0.02	1.19 ± 0.01
4		2.0 (2)	6.5	107.5	25.0	254.62 ± 5.84	36.86 ± 1.18	2.58 ± 0.08	1.35 ± 0.07
5		1.5	7.5 (3)	107.5	20.0	278.92 ± 3.70	39.58 ± 2.22	3.35 ± 0.05	1.46 ± 0.01
6		2.5	7.5	105.0 (1)	25.0	254.48 ± 10.39	43.35 ± 1.88	3.33 ± 0.09	1.29 ± 0.01
7		1.5	6.5	105.0	15.0	216.98 ± 3.52	29.62 ± 0.68	2.11 ± 0.04	1.34 ± 0.11
8		2.0	7.5	110.0	15.0	367.79 ± 24.84	48.75 ± 1.82	3.37 ± 0.09	1.38 ± 0.08
9		2.0	7.0	105.0	20.0	253.30 ± 7.51	36.96 ± 1.80	2.96 ± 0.03	1.43 ± 0.05
3-DG	K_1	6.67	8.07	8.47	8.47	Optimized formulation: $A_1B_1C_2D_2$			
	K_2	7.76	8.44	7.93	7.93				
	K_3	10.45	8.38	8.47	8.47				
	R	3.77	0.31	0.54	0.54				
2,3-BD	K_1	1.02	1.05	1.14	1.45	Optimized formulation: $A_1B_1C_2D_2$			
	K_2	1.13	1.19	1.07	1.07				
	K_3	1.19	1.10	1.11	1.11				
	R	0.16	0.09	0.04	0.39				
MGO	K_1	275.26	272.76	241.50	289.37	Optimized formulation: $A_1B_1C_1D_3$			
	K_2	291.46	288.72	271.27	293.11				
	K_3	294.90	300.14	348.87	279.15				
	R	19.64	27.38	107.60	13.96				
GO	K_1	38.53	36.92	36.69	38.65	Optimized formulation: $A_1B_1C_1D_1$ or $A_1B_1C_1D_2$			
	K_2	40.71	40.19	37.99	40.13				
	K_3	41.67	43.80	46.22	42.14				
	R	12.97	6.59	1.35	1.49				

Table S3. The optimization of MR process parameters for Flonuder condiment formulation in the papain group.

No.		D-xylose addition (%) (A)	pH value (B)	Temperature (°C) (C)	Time (min) (D)	MGO (ng/mL)	GO (ng/mL)	3-DG (ng/mL)	2,3-BD (ng/mL)
1		2.0（1）	7.0（3）	110.0（3）	20（2）	366.34 ± 9.01	42.51 ± 2.35	2.54 ± 0.09	1.35 ± 0.01
2		2.5	6.0（1）	110.0	25（3）	640.11 ± 10.96	55.45 ± 1.99	3.23 ± 0.02	1.59 ± 0.03
3		2.5（2）	7.0	107.5（2）	15（1）	446.11 ± 12.645	49.55 ± 0.46	2.46 ± 0.11	1.19 ± 0.14
4		2.0	6.5 （2）	107.5	25	496.82 ± 8.77	43.80 ± 0.44	2.58 ± 0.8	1.35 ± 0.02
5		3.0（3）	6.0	107.5	20	789.20 ± 13.60	62.24 ± 2.57	3.35 ± 0.07	1.46 ± 0.05
6		3.0	7.0	105.0（1）	25	521.12 ± 7.54	56.44 ± 2.33	3.33 ± 0.14	1.29 ± 0.01
7		2.0	6.0	105.0	15	499.72 ± 15.11	45.98 ± 1.65	2.11 ± 0.06	1.34 ± 0.06
8		3.0	6.5	110.0	15	503.03 ± 5.06	52.22 ± 0.88	3.37 ± 0.12	1.38 ± 0.02
9		2.5	6.5	105.0	20	465.26 ± 14.23	48.21 ± 1.56	2.96 ± 0.07	1.43 ± 0.09
3-DG	K_1	2.41	2.89	2.79	2.72	Optimized formulation: $A_1B_3C_1D_1$			
	K_2	2.88	2.96	2.79	2.97				
	K_3	3.35	2.77	3.05	2.94				
	R	0.94	0.19	0.25	0.25				
2,3-BD	K_1	1.34	1.46	1.35	1.33	Optimized formulation: $A_1B_3C_1D_1$			
	K_2	1.40	1.38	1.33	1.39				
	K_3	1.37	1.27	1.43	1.39				
	R	0.06	0.18	0.11	0.05				
MGO	K_1	452.67	641.15	477.76	505.73	Optimized formulation: $A_1B_3C_1D_1$			
	K_2	498.56	470.91	575.27	511.37				
	K_3	604.99	444.15	503.23	536.35				
	R	152.32	197.00	97.51	30.62				
GO	K_1	44.09	54.71	49.96	49.96	Optimized formulation: $A_1B_2C_1D_1$ or A_1B_3 C_1D_2			
	K_2	51.06	48.12	51.31	50.58				
	K_3	57.06	49.37	50.31	51.45				
	R	12.97	6.59	1.35	1.49				

Table S4. Sensory evaluation indicators for Flounder seafood condiment.

Sensory indicators	Scoring Criteria	Score
Flavour	Aromatic, with the characteristic aroma of flounder.	30
Taste	The flounder is fresh and aromatic, moderately salty	40
Colour	Good lustre, appetising.	20
Condition	Medium consistency, uniform state.	10

Table S5. Sensory scoring criteria for Flounder seafood condiment.

Indicators	Classification standards				
	Very good	Better	Good	Fair	Poor
Flavour	30~26	25~21	20~16	15~11	10~6
Taste	40~36	35~31	30~26	25~21	20~16
Colour	20~17	16~13	12~9	9~6	5~1
Condition	10~9	8~7	6~5	4~3	2~1
